# Editorial: Nitrogen Use Efficiency and Sustainable Nitrogen Management in Crop Plants

**DOI:** 10.3389/fpls.2022.862091

**Published:** 2022-04-11

**Authors:** Nandula Raghuram, Tariq Aziz, Surya Kant, Jianbin Zhou, Susanne Schmidt

**Affiliations:** ^1^School of Biotechnology, Guru Gobind Singh Indraprastha University, Dwarka, India; ^2^International Initiative, New Delhi, India; ^3^Sub-Campus Depalpur Okara, University of Agriculture Faisalabad, Punjab, Pakistan; ^4^Agriculture Victoria, Grains Innovation Park, Horsham, VIC, Australia; ^5^School of Applied Systems Biology, AgriBio, La Trobe University, Bundoora, VIC, Australia; ^6^College of Natural Resources and Environment, Northwest A&F University, Yangling, China; ^7^School of Agriculture and Food Sciences, The University of Queensland, St Lucia, QLD, Australia

**Keywords:** NUE, climate, nitrate, ammonia, nitrous oxide, NOX, policy, reactive nitrogen

## Introduction

Reactive nitrogen (N) impacts all UN sustainable development goals. Nitrogenous fertilizers have enhanced food security and without them, half of our current human population would not be alive. However, reactive N pollution has crossed earth's planetary boundary of sustainability (Steffen et al., 2015), and nitrogenous fertilizers are among its major contributors. The loss of nitrous oxide, ammonia, nitrates, etc. from unused N-fertilizers causes adverse impacts on soil, water, air, climate change, and biodiversity loss. Billions of dollars per annum are also spent on N-fertilizers globally (Sutton et al., 2019). For over two decades, the International Nitrogen Initiative (INI) has been galvanizing scientists and policy makers to overcome this growing challenge from agriculture, fossil fuel burning, and other anthropogenic sources (www.initrogen.org). Its efforts culminated in the recent intergovernmental recognition of this global concern and the adoption of the first ever India-led UN resolution on sustainable nitrogen management (Raghuram and Sharma, 2019; UNEP, 2019; Raghuram et al., 2021; Sutton et al., 2021).

The resolution heralds the global transition from highlighting problems to finding solutions. Some regions/countries of the world have already quantified their N-losses, such as Europe (Sutton et al., 2011), India (Abrol et al., 2017), and Pakistan (Aziz et al., 2021), while others will do their own assessments for informed policy interventions. The solutions for crop N use efficiency (NUE) have to come from the integration of plant biology, agronomy, genetics, and allied disciplines, as there is no single means to improve crop NUE. Open access journals like Frontiers can accelerate this process with frequent calls for submissions on this topic. This collection of research and review articles is not only very timely from this perspective, but is also becoming a series, considering that another call is already open (https://www.frontiersin.org/research-topics/25179/nitrogen-use-efficiency-plant-biology-to-crop-improvement). The uniquely interdisciplinary nature of Frontiers journals and their collaboration on Research Topics complements other subject-wise collections, such as this triennial on plant NUE (https://academic.oup.com/jxb/issue/71/15) and this INI-led focus on environmental aspects (https://iopscience.iop.org/journal/1748-9326/page/Focus_on_Reactive_Nitrogen).

## Interim Options

In the meantime, a lot can be done to improve overall NUE in cropping, based on the lessons learnt so far. Agriculture was invented in Asia over 11,000 years ago; farmers exploited natural biological N fixation in symbiotic legumes to sustainably support human civilization and population growth for millennia (Galloway et al., 2008). The fixed N left over in the soil after a legume crop supports the N needs of the next non-legume crop to a large extent. The restoration of legume-based cropping systems from repeated monocultures or other unsustainable crop-rotations can substantially reduce N-fertilizer demand. In addition, all the available manure, crop waste, and other nutrient-rich wastes ought to be recycled to support agriculture and horticulture to minimize fertilizer dependence.

Similarly, one can expand the use of microbial bio-fertilizers and slow-release chemical fertilizers incorporating urease inhibitors and/or nitrification/denitrification inhibitors, by making them affordable. Further, the agronomic optimization of the 4Rs of nutrient stewardship, i.e., right source, right form, at right time, and with the right method of application will maximize N utilization and minimize N losses. Some of them are aided by leaf color charts or equipment such as chlorophyll meters, aerial spectral sensors, digital photography, fertigation, or other application machinery. These measures may not eliminate fertilizer demand, but they can significantly reduce it by minimizing wastage of resources as well as reducing environmental impact.

## Reorienting Plant Science Research

In whichever form N-inputs are provided to the crop, there is bound to be wastage unless the crop uses most, if not all that is provided. This is essentially a biological problem, which needs to be tackled by understanding and using the phenotype and genotype of N-response and NUE for crop improvement by selection and/or breeding. There is adequate genetic variability for this in many crops (Pathak et al., 2008; Raghuram, 2008; Raghuram and Sharma, 2019). However, the excessive emphasis on yields during the green revolution years meant that crop improvement favored even small increases in yield by ignoring the large increases in inputs, especially of fertilizers. This was further compounded by the conspicuous lack of low N-input screening programs, resulting in reducing efficiencies with increasing yields.

This approach may also have resulted in the loss or rejection of N-use efficient genotypes due to their apparently low N-responsive yield. This calls for revisiting the germplasm and including the landraces and farmers' varieties for screening under low N-input conditions to identify donor genotypes for NUE. This cannot be left to the choices of individual scientists but must be institutionalized through appropriate policies, resources, and other support systems. The lack of such systems may largely explain the delay in scientific breakthroughs toward NUE or any input use efficiency for that matter. After all, it is far more convenient for individuals to work with well characterized “improved” varieties rather than to rediscover things from scratch using landraces.

Nevertheless, the extensive as well as intensive growth of research into the functional biology of N-response and toward crop improvement for NUE in the last couple of decades is impressive. Yet, identifying the most important genes/alleles involved and bringing them together to improve NUE in the field remains a formidable challenge. This collection of articles provides glimpses into some of the latest research trends toward NUE improvement. It may even have set a few new trends, such as in phenotyping and shortlisting/validating candidate genes for NUE. This collection contains 17 original research articles, 3 reviews, 1 opinion, and 1 perspective article. We have categorized them under five headings for summarizing their findings as follows:

## Defining Nue for Today and Tomorrow

Selecting the genotypes with high NUE and designing better N management for them require reliable definitions and indicators. A major problem in this field has been the diversity of definitions and indicators used by agronomists, physiologists, and others. In this context, Congreves et al. provide their perspective on some traditionally ignored indices to improve the concept of NUE. They include widening the range of soil N forms, considering how plants mediate their response to the soil N status, blending agronomic performance with ecosystem functioning, and capturing the synchrony between available N and plant N demand, including the below-ground/root N pools.

## Phenotype, Genotype, and Candidate Genes for Nue

A major hurdle in crop improvement for NUE was the lack of comprehensive characterization of its phenotype and genotype and experimental distinction between N-response and NUE in any crop. Sharma et al. systematically studied 25 common phenotypic parameters in rice, of which 20 were found to be N-responsive. Only eight of them contributed to yield and NUE, of which only six were common to the different N-forms, urea, and nitrate. They also identified 34 genes associated with the NUE phenotype in rice. Kumari et al. complemented this approach with a meta-analysis of N-responsive genes and yield-related genes in rice from literature, using various genetic and bioinformatic tools. They reported a novel approach for hierarchical shortlisting of NUE candidate genes using their associated traits, important physiological processes, quantitative trait loci, and their chromosomal hotspots.

Automated phenotyping methods and phenomics are revolutionizing phenotyping in greenhouses or even in open fields. Here, Wang et al. reported an automated digital image-based phenotyping tool for screening NUE of perennial rye grasses that can be used for other forage crops and for genomic selections. Similarly, canopy cover (CC) is a measurement of the proportion of cropland covered by vertical projection of a crop canopy with the help of digital photography and computerized image analysis. CC has been used earlier in many crops for measuring growth indices, with only few studies focusing on the effect of N and the cultivar relationship. Zhao et al. reported that CC is an easily measured index using a digital camera in winter wheat, but it has many weaknesses and so it could not be used for measuring N- status.

For developing new cultivars with improved NUE, old genotypes with genetic variability could be used. Lupini et al. reported that landraces and ancient genotypes have better vegetative growth and tolerance to both N-stress and water stress. They suggested that ancient durum wheat genotypes are more suitable for sustainable cropping systems in the Mediterranean environment.

## Molecular Physiology of Nue

NUE is a complex polygenic trait spanning many physiological processes such as N uptake, primary assimilation, partitioning, translocation, secondary remobilization, etc., which makes its genetic dissection and engineering very challenging. The et al. reviewed the research related to N-metabolism, transport, and their applications in improving NUE. They highlighted the limitations of earlier targets of genes and processes and discussed other options for the improvement of NUE in plants. Amino acid metabolism is the foundation for both nitrogen uptake efficiency (NUpE) as well as nitrogen utilization efficiency (NutE). It also plays important roles during abiotic stress including, cold, drought, etc., and acclimation to a low N-condition. Genetic engineering of amino acid metabolism has shown some promise in the improvement of crop NUE, especially under low N conditions. Dellero has reviewed the present and future targets of amino acid metabolism for NUE improvement and sustainable agriculture.

The stem is the most important organ for increasing harvest index in cereal crops. Souza and Tavares emphasize in their mini review that a clear understanding of N-regulation of stem development will reveal more genetic tools to alter stem architecture and plant N demand. In poplar, soil N availability plays an important role in the sustainability of high biomass production. For better plant growth and improved NUE, appropriate N application is needed. Hu et al. reported photo respiratory metabolism, polyamines metabolism, and excitation energy allocation in photosystem II as potential regulatory hubs for poplar response to soil N-availability.

## From Transgenic to Genome Editing for Nue

Engineering any multi-genic trait through transgenics can be a challenge, unless one or a few genes account for most of the potential for its improvement. Efforts for transgenic improvement of NUE over the last three decades have met with limited success, mostly due to the trial and error approach in the choice of genes (Raghuram and Sharma, 2019; Madan et al., 2022). However, things seem to be improving, as Chen et al. show here that the co-overexpression of OsNAR2.1 and OsNRT2.3a could increase rice yield, N transport efficiency, N recovery efficiency, and agronomic N use efficiency in rice. Over expression of OsNRT2.3a alone did not show any significant difference in agronomic traits, nitrate uptake, and biomass and N accumulation between transgenic and wild-type lines, but co-overexpression was more effective.

Lysine-histidine-type transporter 1 (OsLHT1) is a key transporter for the uptake of root amino acids and for redistribution of N from source leaves to grain in rice. Using a knockout of this gene in rice, Guo et al. demonstrate its important role in grain yield by the translocation of amino acids from vegetative to reproductive organs. Alanine aminotransferase (AlaAT) has been a focus of NUE transgenics for many years. Here, Tiong et al. reported improved NUE and increased growth and seed yield in wheat, barley, and rice through its overexpression. A comprehensive profiling of genetic and metabolic responses to the overexpression of AlaAT was reported, unraveling the mechanisms and pathways contributing to NUE.

Shi and Tong showed that overexpression of TaLAMP1 affects wheat adaptation to N availability and regulates plant architecture including plant height, spike number, and grain number. Wheat breeding with improved yield and yield response to N-fertilizer and grain N concentration could be achieved by optimizing TaLAMP1 expression. Genome editing is an effective way to manipulate multiple genes/alleles in a single genotype and is expected to take NUE research to the next level, but application of CRISPR/Cas9 could be very challenging in polyploids. Tiwari et al. discussed the prospects for NUE improvement in potato, a tetraploid. They highlighted the major issues related to genome editing in potato and lessons learnt from earlier research in plants from transgenics to CRISPR/Cas9.

## Sustainable N-Management

Agronomic measures and crop management practices must be optimized for every crop to realize its full potential for NUE. Zhang et al. analyzed the effects of traditional fertilization and bag-controlled release fertilizer (BCRF) on the growth and development of roots, shoots, leaves, and fruit quality and yield in peach. They reported that BCRF can improve the utilization efficiency of fertilizer, reduce ammonia volatilization, and enhance fruit yield and quality in peach trees. Naher et al. reported a bio-organic fertilizer as a green technology that reduces synthetic N requirement by 30% and triple superphosphate (TSP) by 100% in rice production, along with improved soil health.

Accurate diagnosis of N uptake and dry matter accumulation is very essential for improvement of NUE. For this purpose, Zhao et al. reported an N diagnostic model to construct N dilution curves under various water and N fertilizer management conditions. In summer maize, Ren et al. used DSSAT simulations to optimize the N application and planting density to maximize yield. They also reported that irrigation, sowing dates, and pest control need to be considered in order to narrow down the observed yield gap. Bhardwaj et al. reported the use of integrated nutrient management to reduce inorganic fertilizer by supplementing it with green manure or grain legume intercropping. They found that organics management provided the most consistent nutrient supply equivalent to or exceeding inorganic fertilizers at several critical stages of growth, especially at tillering and stem elongation.

Although most of the total N uptake by crops occurs in the pre-flowering stage, post-flowering uptake is still important to crop yield and NUE. Mustard and canola have low NUE and water use efficiency (WUE) as compared to other cereals. Riar et al. reported that N supply rate and timing influenced the partitioning of pre- and post-flowering water use in canola and mustard. They highlighted the importance of N uptake and water availability to the expression of NUE, and that NUE may be more sensitive to seasonal conditions than WUE. Heuermann et al. found that as compared to urea, ammonium nitrate promoted N uptake and increased cytokinin synthesis and translocation in oilseed rape.

## Author Contributions

All authors listed have made a substantial, direct, and intellectual contribution to the work and approved it for publication.

## Conflict of Interest

The authors declare that the research was conducted in the absence of any commercial or financial relationships that could be construed as a potential conflict of interest.

## Publisher's Note

All claims expressed in this article are solely those of the authors and do not necessarily represent those of their affiliated organizations, or those of the publisher, the editors and the reviewers. Any product that may be evaluated in this article, or claim that may be made by its manufacturer, is not guaranteed or endorsed by the publisher.

## References

[B1] Abrol Y. P. Adhya T. K. Aneja V. P. Raghuram N. Pathak H. Kulshrestha U.. (2017). The Indian Nitrogen Assessment: Sources of Reactive Nitrogen, Environmental and Climate Effects, Management Options, and Policies. Cambridge: Elsevier.

[B2] Aziz T. Wakeel A. Watto M. A. Sanaullah M. Maqsood M. A. Kiran A. (2021). Nitrogen Assessment: Pakistan as a Case-Study. Academic Press 195. p.

[B3] Galloway J. Raghuram N. Abrol Y. P. (2008). A perspective on reactive nitrogen in a global, Asian and Indian context. Curr. Sci. 94, 1375–1381.

[B4] Madan B. Malik A. Raghuram N. (2022). Crop nitrogen use efficiency for sustainable food security and climate change mitigation, in Plant Nutrition and Food Security in the Era of Climate Change, eds Kumar V. Srivastava A. Penna S. (Elsevier, Academic Press), 47–72. 10.1016/B978-0-12-822916-3.00003-2

[B5] Pathak R. R. Ahmad A. Lochab S. Raghuram N. (2008). Molecular physiology of plant N-use efficiency and biotechnological options for its enhancement. Curr. Sci. 94, 1394–1403. Available online at: https://currentscience.ac.in/Volumes/94/11/1394.pdf

[B6] Raghuram N. Sharma N. (2019). Improving Crop Nitrogen Use Efficiency, in Comprehensive Biotechnology, Vol. 4, ed Moo-Young M. (Pergamon: Elsevier), 211–220.

[B7] Raghuram N. Sutton M. A. Jeffery R. Ramachandran R. Adhya T. K. (2021). From South Asia to the world: embracing the challenge of global sustainable nitrogen management. One Earth 4, 22–27 10.1016/j.oneear.2020.12.017

[B8] Steffen W. Richardson K. Rockström J. Cornell S. E. Fetzer I. Bennett E. M.. (2015). Planetary boundaries: Guiding human development on a changing planet. Science 347:1259855. 10.1126/science.1259855 25592418

[B9] Sutton M. Raghuram N. Adhya T. K. (2019). The nitrogen fix: from nitrogen cycle pollution to nitrogen circular economy, in Frontiers Emerging Issues of Environmental Concern, ed Pinya S. (Nairobi: United Nations Environment Programme), 52–64.

[B10] Sutton M. A. Howard C. M. Erisman J. W. Billen G. Bleeker A. Grennfelt P. (eds). (2011). The European Nitrogen Assessment: Sources, Effects and Policy Perspectives. Cambridge: Cambridge University Press. 10.1017/CBO9780511976988

[B11] Sutton M. A. Howard C. M. Kanter D. R. Lassaletta L. Móring A. Raghuram N.. (2021). The nitrogen decade: mobilizing global action on nitrogen to 2030 and beyond. One Earth 4, 10–14. 10.1016/j.oneear.2020.12.016

[B12] UNEP (2019). United Nations Environment Assembly of the United Nations Environment Programme, Fourth session, Nairobi, 11–15 March 2019. Resolution on Sustainable Nitrogen Management. UNEP/EA.4/L.16. Available online at: https://wedocs.unep.org/bitstream/handle/20.500.11822/28478/English.pdf?sequence=3andisAllowed=y

